# Glycogen storage disease type VI with a novel PYGL mutation: Two case reports and literature review

**DOI:** 10.1097/MD.0000000000025520

**Published:** 2021-04-23

**Authors:** Qian Zhan, Zili Lv, Qing Tang, Li Huang, Xiuqi Chen, Meixiong Yang, Liancheng Lan, Qingwen Shan

**Affiliations:** a Department of Pediatrics; b Department of Pathology, the First Affiliated Hospital of Guangxi Medical University, Nanning, China.

**Keywords:** gene mutation, glycogen storage disease type VI, PYGL

## Abstract

**Rationale:**

Glycogen storage disease (GSD) type VI is a rare disease caused by the inherited deficiency of liver phosphorylase.

**Patient concerns:**

The proband, a 61-month-old Chinese boy, manifested intermittent hematochezia, growth retardation, hepatomegaly, damage of liver function, mild hypoglycemia, and hyperlactatemia. The other patient was a 107-month-old Chinese girl with growth retardation, hepatomegaly, mild hypoglycemia, and hyperlactatemia. In order to further confirm the diagnosis, we conducted a liver biopsy and detected blood samples for their gene using IDT exon chip capture and high-throughput sequencing.

**Diagnoses:**

According to the clinical symptoms, physical examination, laboratory examinations, liver biopsy, and the genetic test finding, the 2 patients were diagnosed GSD VI.

**Interventions:**

They were treated mainly with uncooked cornstarch.

**Outcomes:**

There were 2 mutations of PYGL gene in this pedigree. c.2467C>T (p. Q823X) and c.2178-2A>C occurred both in the proband and his second sister.

**Lessons:**

As a novel mutation, c.2178-2A>C enriches the mutation spectrum of PYGL gene. The different degrees of elevated lactate is an unusual phenotype in GSD VI patients. It is not clear if this is caused by the new mutation of c. 2178-2A > C. Long-term complications remains to be observed.

## 1 Introduction

Glycogen storage disease (GSD) is a class of inherited metabolic disease that affects the synthesis or decomposition of glycogen due to the deficiency of congenital enzymes. According to the type of enzyme deficiency, GSD is classified as 12 types. Glycogen storage disease type VI (GSD VI, MIM 232700), Hers disease, is caused by mutations of the PYGL gene (MIM 613741) encoding hepatic phosphorylase on chromosome 14q21-q22. It predominantly leads to liver injury. Its incidence is about 1/60 000 to 1/85 000, rarer than other types of GSD.^[1–3]^ Here we report 2 patients from a same Chinese family with GSD VI and summarize their clinical symptoms, laboratory examinations, liver biopsy, and genetic test.

## 2 Case presentation

### 2.1 Patient 1

**Figure 1:**
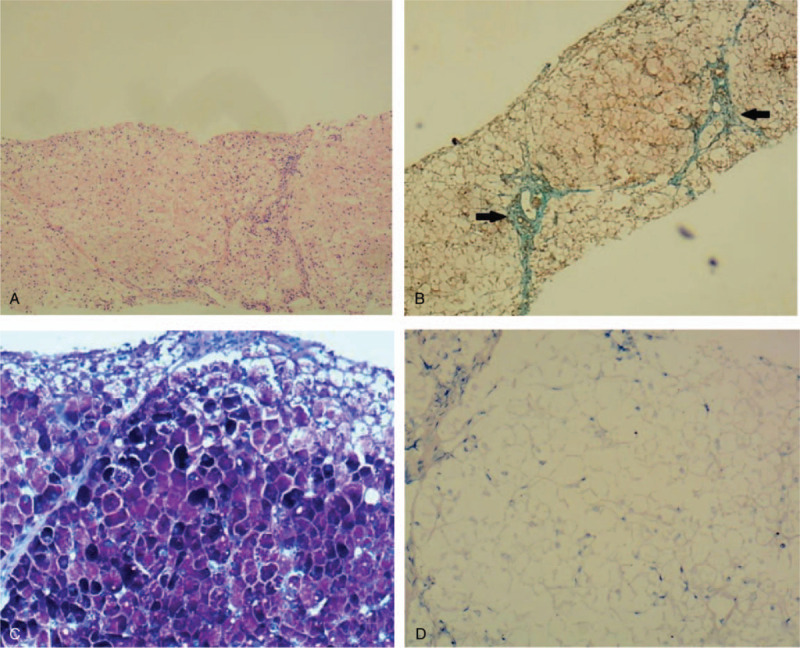
Histological findings of the liver from (A-D) the proband. (A ×40) Hematoxylin–eosin (HE) stain, (B ×40) masson staining, (C ×100) periodic acid-Schiff (PAS) staining and (D ×100) PAS staining after diastase treatment. In the proband specimen, the hepatocytes were (A) arranged neatly and not nodular with (B) fibrosis observed (black arrow). (C) All the hepatocytes were stained strongly by periodic acid-Schiff (PAS), (D) which disappeared following diastase treatment.

A 61-month-old Chinese boy was referred to our hospital for further investigation of intermittent hematochezia, growth retardation, hepatomegaly, and elevation of transaminase. He was the proband in his family. Abdominal distension occurred after birth soon, but no treatment. It was not until there was intermittent hematochezia for 7 months with no abdominal pain, vomiting, and diarrhea that he went to hospital. He was the third child of nonconsanguineous parents. His father was infected with the HBV, while mother and other compatriot with no family history of liver disease as their known. His class grade was the average at school. Physical examination showed growth retardation with a height of 97 cm (<−3SD) and a weight of 9 kg (−3SD to −2SD). There was no special face and no jaundice. It demonstrated hepatomegaly with liver palpable to 7 cm below the right costal margin, whereas the spleen was not palpable. Laboratory data showed liver transaminases (alanine aminotransferase [ALT] 346 U/L, aspartate aminotransferase [AST] 180 U/L), lactate (3.14 mmol/L) and 24-hour urine copper (0.68 μmol/24 h). The fasting blood glucose was slightly reduced after fasting (lowest 3.15 mmol/L). Adrenaline stimulation test had no positive effect to disintegrate glycogen. The biochemistry, blood ammonia, triglycerides, serum copper, ceruloplasmin, hematuria tandem mass spectrometry were within normal values. The autoantibodies of liver disease, hepatitis virus, cytomegalovirus, and epstein-barr virus revealed negative. CT of the abdomen suggested that the liver was tumefacient and the parenchyma density was increased. Since he had hematochezia, enteroscopy was performed. The results showed anal canal inflammation. The liver pathology was compatible with GSD (Fig. 1). Finding showed hepatic lobular structures, neatly arranged hepatocytes and punctate necrosis. Strongly positive was demonstrated in periodic acid-Schiff stain (PAS) in hepatocytes without diastase treatment, while PAS disappeared following diastase treatment. Moreover, masson staining showed more collagen fibroplasia in the slightly distensible portal area. The Ishak score was 3 for inflammation and 3 for fibrosis. To detect the reason for those differences, molecular genetic testing was performed in all members of this family using IDT exon chip capture and high-throughput sequencing by Kindstar Global. The boy was diagnosed GSD and treated with uncooked cornstarch (2 g/kg, 6 hours intervals, taken between meals, before bedtime and at night), which serves as a slow release carbohydrate to prolong euglycemia between meals.^[4]^

He was followed up with regular visits in our hospital. He still had repeated the presence of blood in stools 1 month later, but hepatomegaly reduced to 3.5 cm below the right costal margin. He suffered from acute upper respiratory infection at follow-up visit. Laboratory results showed ALT 97 U/L, AST 91 U/L, blood glucose 3.46 mmol/L, and lactate 1.68 mmol/L. Glutathione (0.1 g 3 times daily) and mesalamine (0.5 g once a day) were used in planning treatments. At 15 months follow-up, various data of the boy had changed (Table 1). By the 15th month of return visit, the boy still occasionally presented with presence of blood in stools. Hepatomegaly was reduced to 2.5 cm below the right costal margin. The transaminases and lactate returned to normal. The fasting blood glucose was slightly reduced (3.51 mmol/L).

**Table 1:** Clinical data.

Age (mo)	Height (cm)/growth evaluation (SD)	Weight (kg)/growth evaluation (SD)	Liver size (cm)	Glu (mmol/L)	ALT (U/L)	AST (U/L)	Lac (mmol/L)
61	97	15	7	3.42	59	57	3.41
	<−3SD	−3SD to −2SD					
62	98	15	3.5	3.46	97	91	1.68
	<−3SD	−3SD to −2SD					
64	–	–	3.5	3.97	27	43	2.37
65	–	–	3	4.19	17	33	2.74
70	–	–	3	4.43	19	38	2.91
75	108	16.5	2.5	3.25	92	252	2.26
	−3SD to −2SD	−3SD to −2SD					
76	108	16.5	2.5	3.51	22	43	2.22
	−3SD to −2SD	−3SD to −2SD					

### 2.2 Patient 2

**Figure 2:**
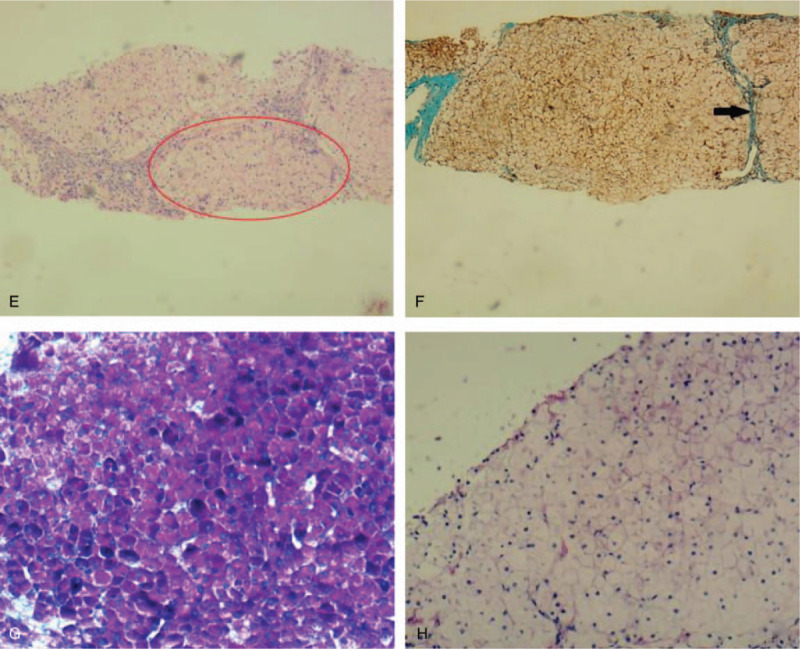
Histological findings of the liver from (E-H) the older sister of proband. (E ×40) Hematoxylin–eosin (HE) stain, (F ×40) masson staining, (G ×100) periodic acid-Schiff (PAS) staining and (H ×100) PAS staining after diastase treatment. In the specimen, (E) Hepatocytes were arranged mostly neatly, but nodular pattern in small areas such as circled parts (red circle). (F) Fibrosis was observed (black arrow). (G) All the hepatocytes were stained strongly by periodic acid-Schiff (PAS), (H) which obviously receded following diastase treatment.

A 107-month-old Chinese girl who was the second child of this family was likely to be a GSD VI due to the abnormal gene. She had no overt clinical manifestation, such as abdominal distention, hepatomegaly, except growth retardation and occasionally presence of blood in stools what they did not value. Her academic achievement is below the average. Physical examination suggested growth retardation with a height of 125 cm (−2SD to −1SD) and a weight of 22 kg (−2SD to −1SD). The liver was swollen to 4 cm below the right costal margin without abdominal distention. Vegetations existed in the anus at 6 o’clock. Laboratory examinations revealed mildly reduced fasting blood glucose (lowest 3.66 mmol/L). Adrenaline stimulation test had no positive effect to disintegrate glycogen. Alpha-fetoprotein and liver transaminases were within reference ranges. She had hematochezia and enteroscopy was performed. The results were normal. The liver pathology was compatible with GSD (Fig. 2). Hepatic lobular structures were present, with hepatocytes arranged neatly in most of the areas. Small areas of hepatocytes were arranged in a nodular pattern. The masson staining had fibrosis observed around portal areas. PAS in hepatocytes without diastase treatment was strongly positive, while PAS obviously receded following diastase treatment. It was also worth noting that masson staining showed more collagen fibroplasia in the distensible portal area. The Ishak score was 4 for inflammation and 3 for fibrosis. She was treated with uncooked cornstarch (2 g/kg, 6 hours intervals, taken between meals, before bedtime and at night). For 12 months of follow-up visit, various data of the girl had changed (Table 2). By the 12th month of return visit, the girl had no obvious symptoms. The liver size changed from 4 cm to 2.5 cm below the right costal margin. The liver transaminases returned to normal. The fasting blood glucose was still mildly reduced (3.83 mmol/L). Lactate was normal (2.29 mmol/L).

**Table 2:** Clinical data.

Age (mo)	Height (cm)/Growth evaluation (SD)	Weight (kg)/Growth evaluation (SD)	Liver size (cm)	Glu (mmol/L)	ALT (U/L)	AST (U/L)	Lac (mmol/L)
107	125	22	4	3.66	52	40	–
	−2SD to −1SD	−2SD to −1SD					
108	–	–	4	4.57	17	22	1.75
113	–	–	4	3.72	38	33	2.69
118	–	–	2.5	3.45	18	23	1.82
119	135	30.5	2.5	3.83	22	33	2.29
	−1SD∼Median	−1SD∼Median					

**Figure 3:**
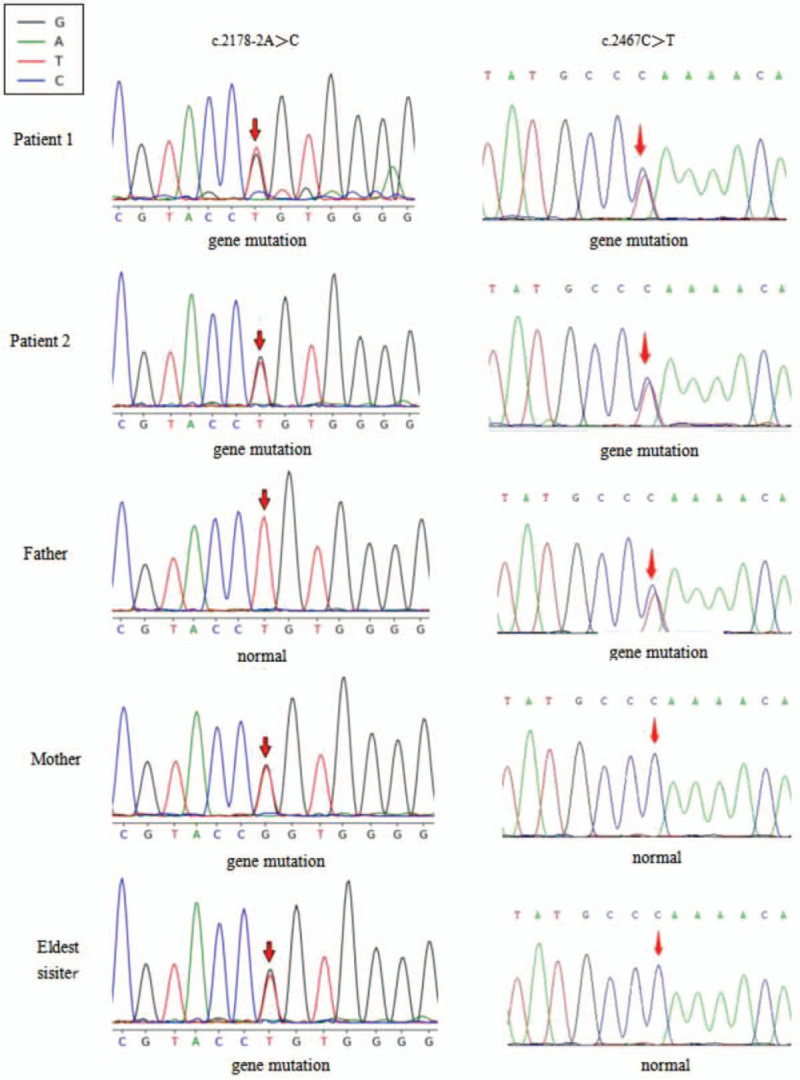
The father had c. 2467C > T (p.Q823X) mutation, without c. 2178-2A > C mutation. The mother and her eldest child both had c. 2178-2A > C mutation, without c. 2467C > T (p.Q823X) mutation. The 2 cases had both c.2467C>T (p.Q823X) and c.2178-2A>C.

**Figure 4:**
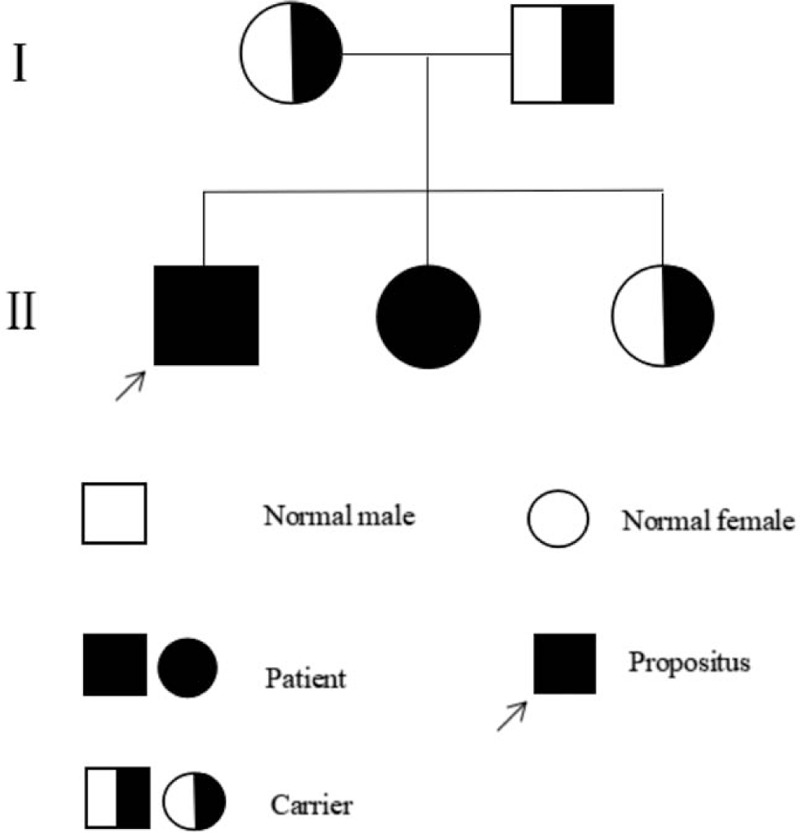
I: From left to right, shown are the mother and father in this family. II: From left to right, shown are the proband, the second sister and the eldest sister respectively. The parents and the eldest sister were carriers of gene mutation. The proband and his second sister were patients.

The results of gene suggested that both 2 cases inherited compound heterozygous mutations on the PYGL gene, including c.2467C>T (p.Q823X) on exon 20 (chr14:51372187) from father and c.2178-2A>C on exon 18 (chr14:51375675) from mother. Based on the clinical manifestation, physical, laboratory, and pathology, they were diagnosed as GSD VI. Their eldest sister, the first child of parents, inherited c.2178-2A>C heterozygous mutation from mother, but no c.2467C>T mutation (Fig. 3). The father, mother and eldest sister were disease-free with single heterozygotes. They belonged to carriers of gene mutation. In summary, the inheritance of the mutation in this family accords with the law of Mendelian inheritance and the mode of inheritance accords with the characteristics of autosomal recessive inheritance (Fig. 4).

## 3 Discussion

The relevant literature was searched in CNKI and PubMed. A total of 22 related articles with relatively complete data were found. The first international report of the disease was reported by Hers in 1959.^[5]^ Atsushi Ogawa indicated a case of GSD VI complicated by focal nodular hyperplasia.^[6]^ Including this report, about 42 cases of GSD VI had been reported.^[1,2,6–18]^ Twenty three cases were male and 19 cases were female. Thirty four cases were at abroad and 8 cases in China. The minimum age for symptoms was 1 month-old. The maximum age of diagnosis is 31 year-old. Main symptoms were vomiting, abdominal distension, feeding difficulties, glycogenic hepatomegaly, growth retardation, and short stature. Most of them were due to hepatomegaly and growth retardation. In 42 cases of GSD VI patients, hepatomegaly (97.6%, 41/42), elevated transaminases (71.4%, 30/42), growth retardation (38.1%, 16/42), hyperlipidemia (35.7%, 15/42), hypoglycemia (23.8%, 10/42), and abdominal distension (14.3%, 6/42). GSD VI with significant hepatomegaly and elevated transaminases could be early diagnosed in infancy. Patients with mild or atypical clinical symptoms were likely to be misdiagnosed or delayed. To date, the Human Gene Mutation Database (http://www.hgmd.cf.ac.uk/ac/index.php) has reported around 50 mutations in PYGL associated with GSD VI.^[17]^ There are missense mutations, nonsense mutations, splicing mutations, synonymous mutations, open reading frame changes, etc. Missense mutations account for the majority.

Glycogen storage disease type VI is a genetic disorder of sugar metabolism due to hepatic phosphorylase deficiency. At present, the PYGL gene is the only gene clearly associated with GSD VI.^[19]^ Classic manifestations include growth retardation, hepatomegaly, mild hypoglycemia, ketosis, hyperlipidemia, elevated transaminases, and generally normal lactate and uric acid.^[20]^ Some researchers had found that liver glycogen accumulated excessively over time to increase the risk of liver injury, inflammation and fibrosis by constructing PYGL gene mutation mouse model.^[19]^ In this report, there were 2 mutations of PYGL gene in this pedigree. c.2467C>T (p. Q823X) and c.2178-2A>C occurred both in the proband and his second sister. c.2467C>T (p.Q823X) had been reported.^[7]^ It was a nonsense mutation. The population frequency was 0.00006 in the database of 1000 Genomes Project, ESP6500 and ExAC. According to the 2015 American Society of Medical Genetics and Genome (American College of Medical Genetics, ACMG), the mutation was classified as probable disease (PM2+PM3+PP4+PP5). It was worth mentioning that a novel mutation, c.2178-2A>C, never been reported and caused a splicing mutation. c.2178-2A>C was classified as a pathogenic mutation (PVS1+PM2+PP4) according to the ACMG, resulting in forming incorrect mRNA molecule.

The proband had intermittent hematochezia and anorectal inflammation, so inflammatory bowel disease was suspected. Mesalaxin was applied locally on the intestinal mucosa to reduce the production of leukotriene and scavenge free radicals.^[21,22]^ Anti-inflammatory treatment of mesalaxin was effective because of improved hematochezia. In addition to intermittent hematochezia, the proband also experienced growth retardation, hepatomegaly, damage of liver function, mild hypoglycemia and hyperlactatemia. His sister showed growth retardation, hepatomegaly, mild hypoglycemia, and hyperlactatemia. The same PYGL gene mutation occurred in both patients, but the phenotypes were not identical. This may be related to the difference of gene expression and penetrance. Lactate concentration ≥ 2 mmol/L without acidosis was called hyperlactatemia.^[23]^ They had different degrees of elevated lactate. The liver plays an important role in the clearance of lactate, with high clearance but also speediness. Lactate clearance decreased after hepatic impairment. The gluconeogenic pathway of GSD VI was intact. Glucose-6-phosphate and pyruvic acid accumulates by the bypass metabolism of glucose-6-phosphate stimulated by hypoglycemia. Pyruvate metabolism produces excess lactic acid. Regular follow-up of the 2 patients were without hypoxia or severe infection. Lactate level did not appear to be significantly correlated with liver transaminases or blood glucose. This unusual phenotype was not encountered in GSD VI patients. Again, the cause for this unusual phenotype is currently unknown. Is it possible to speculate that hyperlactatemia may be an expressive effect caused by the new mutation of c. 2178-2A > C?

After more than 1 year of follow-up, the blood glucose and liver transaminases levels of the 2 patients in this report were controlled moderately at present, at the same time, catch-up growth was partly achieved. Growth retardation, elevated transaminases, and hypoglycemia could be improved after dietary treatment with uncooked cornstarch. The results were consistent with those reported by LUO XM.^[17]^ The patient's family believed that they could effectively manage the disease with regular visits and treatment in the future. The second sister had developed nodular arrangement of hepatocytes at the time of diagnosis, however, with rather short-term follow up, it is unclear at present whether there would be other long-term complications.

## 4 Conclusion

Here we present 2 GSD VI siblings with a compound heterozygous mutation including c. 2467C > T (p.Q823X) and c. 2178-2A > C (splicing). As a novel mutation, c.2178-2A>C enriches the mutation spectrum of PYGL gene. For hereditary diseases, genetic test is conducive to improve the understanding and guide the patient for genetic counseling.

## Author contributions

**Conceptualization:** Qingwen Shan

**Data curation:** Qian Zhan, Meixiong Yang, Liancheng Lan

**Formal analysis:** Qian Zhan

**Methodology:** Liancheng Lan

**Resources:** Zili Lv

**Software:** Xiuqi Chen

**Supervision:** Qing Tang

**Visualization:** Li Huang

**Writing – original draft:** Qian Zhan

**Writing – review & editing:** Qingwen Shan

## Corrections

PYGL gene was spelled incorrectly as PGYL gene in two places in the second paragraph of the discussion section. This has been corrected.
